# Research and experimental verification of the molecular mechanism of berberine in improving premature ovarian failure based on network pharmacology

**DOI:** 10.1080/21655979.2022.2062104

**Published:** 2022-04-14

**Authors:** Wu Xue, Fan Xue, Tao Jia, Ai Hao

**Affiliations:** a Graduate School, Jinzhou Medical University, Jinzhou, Liaoning, China; b Key Laboratory of Follicular Development and Reproductive Health of Liaoning Province, Jinzhou Medical University, Jinzhou, Liaoning Province, China; c Department of Obstetrics and Gynecology, The Third Affiliated Hospital of Jinzhou Medical University, Jinzhou, Liaoning, China; d Department of Obstetrics and Gynecology, The First Affiliated Hospital of Jinzhou Medical University, Jinzhou, Liaoning, China

**Keywords:** Premature ovarian failure (POF), berberine (BBR), network pharmacology, granulosa cells (GCs), in vivo experiment, in vitro experiment

## Abstract

Based on the research methods of network pharmacology, this study analyzed the improvement effect of berberine (BBR) on premature ovarian failure (POF) and its molecular mechanism. Carry out GO and KEGG enrichment analysis by R language to obtain the potential targets and pathways of BBR in the improvement of POF. Use SD rats and ovarian granulosa cells (GCs) for experimental verification. ELISA was used to measure the content of related hormones in the serum, CCK-8 was used to measure cell viability, western blot was used to measure the content of the target protein in the ovaries and GCs, and q-RT-PCR was used to detect the expression of the target genes in the ovaries and GCs. Predicted by network pharmacology: PTEN, AKT1, FoxO1, FasL, and Bim are the targets with the highest relative correlation between BBR and POF. The results of experiments show that the treatment of low and medium doses of BBR can increase the ovarian index of rats; BBR can increase the levels of Estradiol (E_2_) and Anti-Mullerian hormone (AMH) in the serum of rats and reduce the levels of Follicle stimulating hormone (FSH) and Luteinizing hormone (LH). BBR can increase the cell viability of GCs; BBR can inhibit the PTEN/AKT1/FoxO1 signaling pathway and its phosphorylation level and reduce the expression of Fas/FasL and Bim mRNA. Overall, BBR can promote the ovarian to maintain normal hormone levels, protect GCs, and enhance the function of POF.

## Graphical abstract

**Figure:**
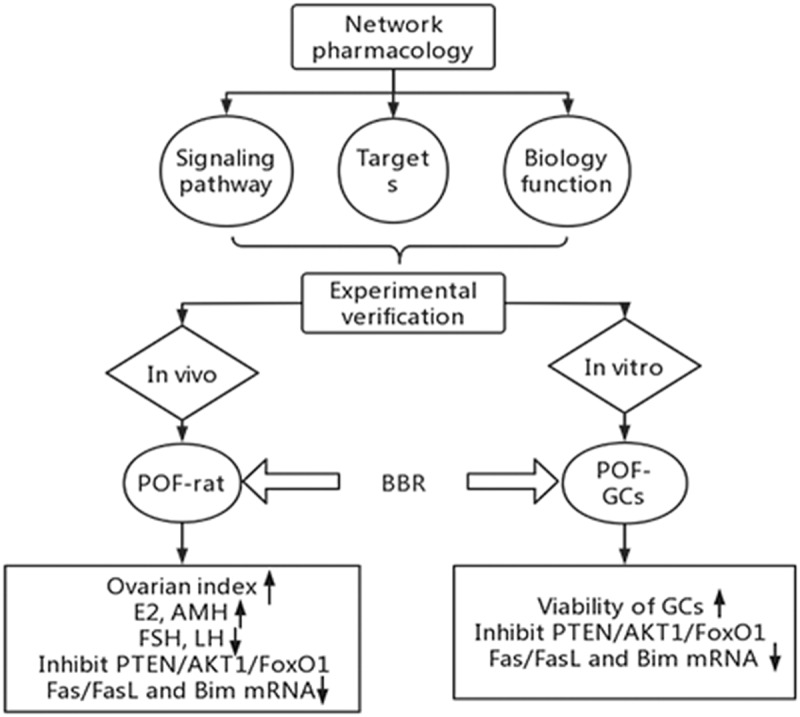

## Introduction

Premature ovarian failure (POF), also known as primary ovarian insufficiency (POI), is a disease characterized by female ovarian reserve failure before the age of 40 and accompanied by high gonadotropin disorders. Mainly manifested as estrogen deficiency and anovulation, primary or secondary amenorrhea, infertility, sex steroid hormone deficiency, and elevated gonadotropin [1]. The pathogenesis and causes of POF are complex. The currently known causes of POF include chromosomal defects, radiation, and drug damage, autoimmune diseases, etc., but the cause of 75%-90% of cases is still unknown [2]. At present, the primary clinical treatment for POF is estrogen-progesterone replacement therapy, but which has the problem of withdrawal reaction, and long-term use may also increase the risk of endometrial cancer and ovarian cancer [3,4].

Berberine (BBR) is an isoquinoline alkaloid extracted from natural medicines such as berberine, three needles, and cork. BBR has significant effects in antibacterial, anti-tumor, hypolipidemic, anti-diabetic, improvement of polycystic ovary syndrome (PCOS), and improvement of autoimmunity. Research has shown that BBR can inhibit the level of inflammatory factors in the GCs of patients with PCOS, increase the levels of AMPK (Adenosine 5‘-monophosphate (AMP)-activated protein kinase) mRNA and IRS-1 (insulin receptor substrate 1) mRNA, and reduce the level of mTOR (mammalian target of rapamycin) mRNA [5], and can activate the PI3K/AKT (phosphatidylinositol 3-kinase/ protein kinase B) signaling pathway and regulate the MAPK signaling pathway [6], so that can achieve the prevention and treatment of PCOS and the effect of protection of GCs. It can be seen that BBR can reverse the oxidation and damage of GCs. GCs provide necessary growth signals and nutrients for oocyte maturation. The state of GCs is critical to the development of follicles. Reducing the apoptosis of GCs and repairing the original follicle reserve pool are the primary mechanisms for restoring ovarian function [7]. Therefore, protecting GCs is also pivotal in improving and preventing POF, but no research shows that BBR can improve POF. This study will explore the potential mechanism of BBR to enhance and prevent POF.

Network pharmacology is a method of constructing drug-target-pathway networks to study the mechanisms of the drug by mapping drug targets to specific biological nodes. Network pharmacology is widely used in traditional Chinese medicine research, including discovering targets, screening active compounds, evaluating toxicity, mechanism research, and quality control research [8]. Network pharmacology analysis can obtain the effects and toxicity of drugs on specific targets and core networks. Network pharmacology provides theoretical support for the transformation of traditional Chinese medicine from empirical medicine to evidence-based medicine. This study will apply the network pharmacology method to analyze the potential mechanism of BBR in improving the POF and conduct in vivo experiments to verify it.

We hypothesized that the network pharmacological analysis could provide us with the targets, pathways, and biological functions of BBR in improving POF. Through in vivo and in vitro experiments, we could verify the potential mechanism of BBR in improving the POF. Provides the basis for the development of drugs for the treatment of POF. Provides a new direction for the wide application of BBR in clinical treatment.

## Materials and methods

### Network pharmacology analysis

#### Screening of potential targets for diseases and drugs

We selected three databases to extract and screen the potential targets of BBR. The three databases include The Comparative Toxicogenomics (CTD) database, traditional Chinese medicine systems pharmacology (TCMSP) database, and STITCH database. CTD (http://ctdbase.org/) is a database that provides manually curated chemical–gene/protein interactions, chemical–disease, and gene-disease relationships. These data are integrated with functional and pathway data to aid in developing hypotheses about the mechanisms underlying environmentally influenced infections. TCMSP (https://tcmspw.com/tcmsp.php) is a unique systems pharmacology platform of Chinese herbal medicines that captures the relationships between drugs, targets, and diseases [9]. STITCH (http://stitch.embl.de/) is a database of known and predicted interactions between chemicals and proteins. The interactions include direct (physical) and indirect (functional) associations; they stem from computational prediction [10]. The potential targets of POF are extracted and screened by The Human Gene (GeneCards) Database. GeneCards (https://www.genecards.org/) is a database that provides comprehensive information on all annotated and predicted human genes [11]. Then the targets of BBR and POF are intersected to obtain the targets of POF and BBR.

#### Protein-protein interaction (PPI) network construction

The targets of POF and BBR were entered into the String database to construct a PPI network. String (https://string-db.org/) is a database of known and predicted protein-protein interactions. The interactions include direct (physical) and indirect (functional) associations [12]. Then input the data obtained from the String database into Cytoscape v3.7.1 [13] to visualize the PPI network.

#### Topological analysis and the core target screening

Topological analysis of PPI network through Cytoscape’s plug-in CytoNCA. The three parameters of degree centrality(DC), closeness centrality (CC), betweenness centrality (BC) are used to analyze the topological properties of each node in the interactive network. After screening the core targets, construct the PPI network of the core targets.

#### GO and KEGG enrichment analysis

The clusterProfiler package of R language is used for GO enrichment analysis and KEGG enrichment analysis. GO enrichment analysis is the analysis of the enrichment level of proteins and genes from the three aspects of biological process (BP), cellular component (CC), and molecular function (MF) [14]. Kyoto Encyclopedia of Genes and Genomes (KEGG) is a database resource for understanding high-level functions and utilities of the biological system, such as the cell, the organism, and the ecosystem, from molecular-level information, especially large-scale molecular datasets generated by genome sequencing and other high-throughput experimental technologies [15].

#### Drug-target-pathway network construction

Construct the drug-target-signal pathway network with the information of drugs, targets, and signal pathways. And import the info into Cytoscape to visualize the drug-target-pathway network. In this network, different colored “nodes’ represent protein targets, drugs, and signaling pathways. ‘Edge’ means the interaction between the drug, the target, and the signal pathway.

### In vivo experiment

#### POF animal model construction and grouping

Select female SD rats weighing 250 g (purchased from Shenyang Changsheng Biological Co., Ltd.), reared in a room at 21 ~ 26°C, light for 12 h, and eat freely. It has been approved by the Experimental Animal Ethics Committee of Jinzhou Medical University (Issue NO. 2021016) (see Figure S1). After three days of adaptive feeding, vaginal smears were performed, and SD rats with regular estrous cycles were selected to construct POF animal models. On the first day, a dose of 50 mg/kg Cyclophosphamide (purchased from MACKLIN China) was injected intraperitoneally, and then a dose of 8 mg/kg Cyclophosphamide was continuously injected intraperitoneally for 14 days to establish a POF disease model rat [16]; The blank control group (group A) was injected intraperitoneally with the same dose of normal saline, and other conditions were the same as the model group. Vaginal smears seven days after the completion of the model, and crystal violet staining is used to test whether the model is successful, and the estrus cycle is disordered as the model is successful [17].

The POF disease model rats were randomly divided into four groups with six rats in each group, namely the model group (group B), the low-dose group (group C), the medium-dose group (group D), and the high-dose group (group E); Similarly, 6 rats whose vaginal smears showed normal estrous cycle were selected to form group A. At 9 o’clock every day, the blank control group (group A) and the model group (group B) were given saline intragastrically; the low-dose group (group C), the middle-dose group (group D), and the high-dose group (group E) were assigned respectively of BBR (purchased from Shanghai Ryon Biological Technology Co., Ltd.) at doses of 100 mg/kg, 200 mg/kg, and 400 mg/kg; continuous gavage for 28 days.

#### ELISA Assay

After the gavage is finished, fast but drink water. On the next day, phenobarbital anesthesia; Take 5 ml of blood from the abdominal aorta and collect it in a 15 ml centrifuge tube. After standing for 30 min, centrifuge at 3000 r/min for 15 min, aspirate the upper layer of serum and dispense it. The experimental operation was performed strictly by the purchased corresponding rat ELISA kit (purchased from Shanghai Enzyme-linked Biotechnology Co., Ltd.). The microplate reader measured the absorbance (OD) value at a wavelength of 450 nm [18], and the levels of E_2_, FSH, LH, and AMH in the serum of each group of samples were calculated by the standard curve.

#### Ovarian index measurement

After blood collection, the rat’s bilateral ovaries were taken, and the ovarian index (wet weight of the ovaries /body weight) was measured. After peeling the surrounding fatty tissue, the ovaries were placed in a cryotube and stored in a refrigerator at −80°C for subsequent experiments.

#### Western blot was used to detect the protein content of PTEN, P-AKT1, AKT1, P-FoxO1, and FoxO1 in the ovaries of rats

Take the frozen rat ovaries, add an appropriate amount of protein lysate, extract the total protein and use the protein quantification box for protein quantification. After electrophoresis on SDS polypropylene gel, transfer to polyvinylidene fluoride (PVDF) membrane. After the primary and secondary antibodies (purchased from ABclonal Technology Co., Ltd. And Boster Biological Technology Co., Ltd.) are incubated, use the ECL Chemiluminescence kit (purchased from BEIJING DINGGUO CHANGSHENG BIOTECHNOLOGY Co., Ltd.) for development [19]. Perform quantitative analysis of the target band of the protein and normalize the target protein content. Process the image.

#### Detect the expression of Fas/FasL and Bim mRNA by q-RT-PCR

The operation steps are carried out according to the kit instructions (purchased from Vazyme Biotech Co., Ltd.). Extract the total RNA of each group of ovaries, reverse transcribed cDNA, and use it as a template for PCR amplification. The amplification conditions are: 95°C pre-denaturation 30s, 95°C, 10s → 60°C, 30s, 40 cycles in total. The internal reference is GAPDH, and the relative content of the initial cDNA of the target gene in the sample is expressed by 2^−ΔΔCt^ [20]. The primer sequences (purchased from BEIJING DINGGUO CHANGSHENG BIOTECHNOLOGY Co., Ltd.) are shown in (see Table 1).

**Table 1.** Primer sequence

Gene	Upstream primer	Downstream primer
FAS	5’-TATCACTGCACCTCGTGTGG-3’	5’-GTTGCCTCTTCCGGTACCTT-3’
FASL	5’-CCTTCCAGGGTGGGTCTACT-3’	5’-GTGCCTCAAACACCCCTCTT-3’
BIM	5’-ACCAAGACCCAGTCGCATAG-3’	5’-GTGCTACCGACATGCACCAA-3’
GAPDH	5’-AGTGCCAGCCTCGTCTCATA-3’	5’-ATGAAGGGGTCGTTGATGGC-3’

### In vitro experiment

#### Primary culture and identification of ovarian granulosa cells

The 21-day SD female rats were intraperitoneally injected with pregnant horse serum (PMSG, 60IU/mouse) for 24 hours and then sacrificed by neck removal. The bilateral ovaries were quickly removed, and the follicles were pierced with a 1 ml syringe needle after peeling off under a stereomicroscope [21]. Collect GCs, inoculate the extracted cells into DMEM/F12 (Dulbecco’s Modified Eagle Medium/Nutrient Mixture F-12) culture system (DMEM/F12 + 10%FBS (Fatal bovine serum) +1% Penicillin) at an appropriate concentration, and use immunofluorescence [22] to detect the FSHR (follicle-stimulating hormone receptor) protein on the surface of GCs.

#### Cell grouping and administration

The cells are divided into five groups: blank control group (group A), model group (group B), low-dose group (group C), medium-dose group (group D), and high-dose group (group E). Except for the blank control group, the other groups were added 4ug/ul cyclophosphamide to make the model. In contrast, the low, medium, and high dose groups were added with 30, 60, and 90 umol/L BBR hydrochloride, respectively, and the blank control group was added with the same dose of Phosphate buffer saline (PBS).

#### CCK-8 to determine the cell viability of GCs

Inoculate the cells into a 96-well plate. When the cells grow to 60%-70%, add 10ul CCK-8 (purchased from BEIJING DINGGUO CHANGSHENG BIOTECHNOLOGY Co., Ltd.) solution to each group of cells after 24 hours of treatment factors, and continue to incubate at 37°C for 2 hours. Use a microplate reader to measure at 450 nm wavelength [23]. Cell viability = (A(adding medicine)-A(blank))/(A(0 adding medicine)-A(blank)).

#### Western blot was used to detect the protein content of PTEN, P-AKT1, AKT1, P-FoxO1, and FoxO1 in the GCs

Part of the primary GCs was inoculated into a 6-well plate. After the cells grew to 60%-70%, the treatment factors were added to each group of cells for 24 hours, and then the total protein was extracted, and the protein was quantified. After electrophoresis on SDS polypropylene gel, transfer to polyvinylidene fluoride (PVDF) membrane. After the primary and secondary antibodies are incubated, use the ECL Chemiluminescence kit for development [19]. Perform quantitative analysis of the target band of the protein and normalize the target protein content. Process the image.

#### Detect the expression of Fas/FasL and Bim mRNA in the GCs by q-RT-PCR

Part of the primary GCs was inoculated into a 6-well plate. After the cells grew to 60%-70%, the total RNA of each group of cells was extracted according to the kit instructions after adding the treatment factors for 24 hours, reverse transcribed cDNA, and using it as a template for PCR amplification. The amplification conditions are: 95°C pre-denaturation 30s, 95°C, 10s → 60°C, 30s, 40 cycles in total. The internal reference is GAPDH, and the relative content of the initial cDNA of the target gene in the sample is expressed by 2^−ΔΔCt^ [20].

### Statistical analysis

SPSS 26.0 (IBM Corp, Armonk, NY, USA) was adopted to analyze the experimental data. GraphPad Prism version 8.0.2 was adopted to make the figures. The data were expressed as mean ± standard deviation (SD). The t-test was adopted for comparison between two groups, and One-Way analysis of variance (ANOVA) was adopted for comparison among multiple groups. The *P* value was obtained from the two-tailed tests, and a value of *P* < 0.05 was considered to be statistically significant.

## Result

In this study, the effect of BBR in improving POF and its molecular mechanism are explored from two parts, which provides a new basis for the development of POF therapeutic drugs. The first part applies the analytical methods of network pharmacology for data mining, and the second part uses in vivo and in vitro experiments for verification. We suspect that through the analysis of network pharmacology, the corresponding targets, pathways and biological effects of BBR against POF can be obtained and verified by experiments.

### Potential targets for diseases and drugs

**Figure 1.**
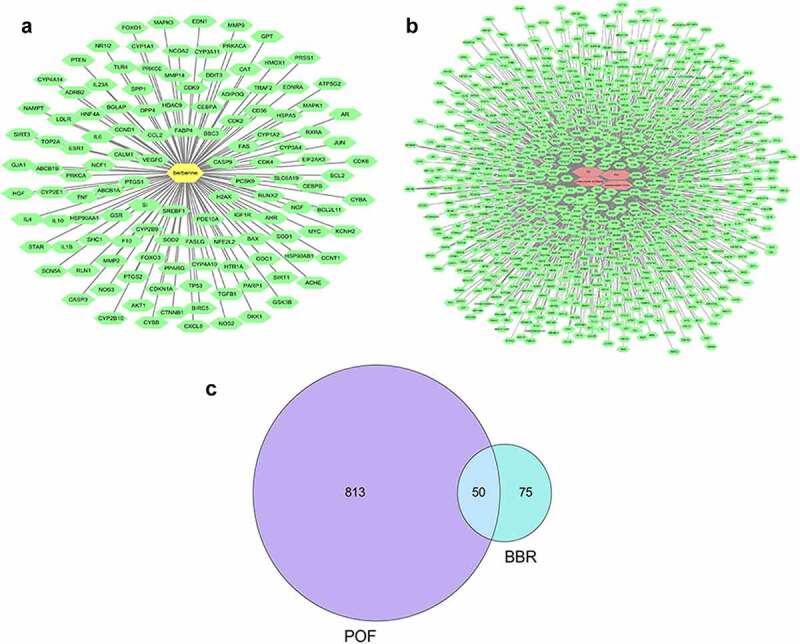
(a) Drug-target diagram; Green dots represent the targets of BBR; Yellow dots represented BBR. (b) Disease-target diagram.; Green dots represent the targets of the disease; Red dots represent the disease. (c) Venn diagram of the intersection of disease target and drug target.

Use ‘berberine’ as a keyword to search and filter in the three databases. 250 targets were obtained in the CTD database, and 104 targets were obtained with ‘interactions>1’ as the screening condition. 17 targets were obtained in the TCMSP database. Get 10 targets in the STITCH database. Remove the duplicate targets to get a total of 125 targets (see Figure 1)). Search for disease targets in the GeneCards database. Use ‘premature ovarian failure’ as a keyword to obtain 643 targets; Use ‘primary ovarian insufficiency’ as a keyword to get 645 targets; Use ‘POF’ as a keyword to obtain 215 targets, and use ‘POI’ as a keyword obtained 227 targets. After removing the duplicate targets, a total of 863 related targets were obtained (see Figure 1)). Then screen out the target of POF and BBR (see Figure 1)).

### PPI network

**Figure 2.**
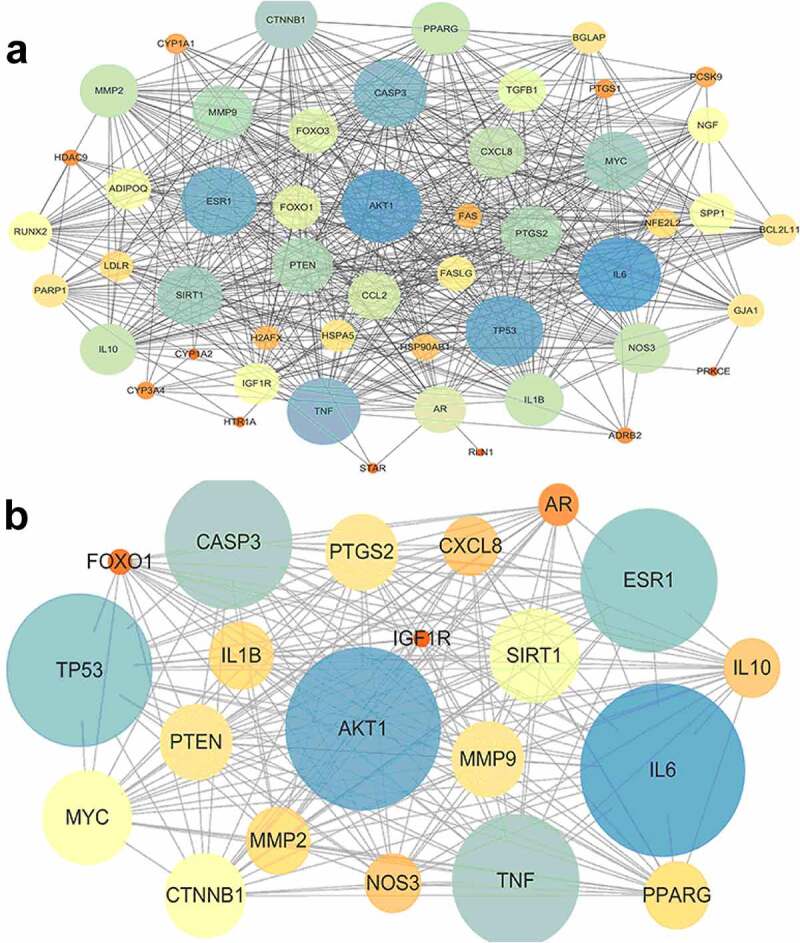
(a) Protein-protein interaction network; The size and color of the target in the PPI network represent the degree of importance in the role. The larger the target, the bluer the color, the more influential the target is. (b) Core protein-protein interaction network; The size and color of the target in the PPI network represent the degree of importance in the role. The larger the target, the bluer the color, the more influential the target is.

Enter these potential targets into the String database to obtain PPI network information and enter the data into Cytoscape for visualization. The PPI network contains 50 nodes and 1094 edges (see Figure 2)). The size and color of the target in the PPI network represent the degree of importance in the role. The larger the target, the bluer the color, the more influential the target is.

### Topological analysis and core targets

Use CytoNCA, a plug-in of Cytoscape, to analyze the topology of the PPI network. According to DC≥24 BC≥17.251 CC≥0.649, 21 core targets were screened out. Enter these core targets into the String database to obtain the core PPI network information (see Table 2) and visualize it (see Figure 2)).

**Table 2.** Core targets

name	Degree	Betweenness	Closeness	name	Degree	Betweenness	Closeness
IL6	41	119.44	0.86	MMP9	31	17.49	0.73
AKT1	40	81.91	0.84	IL1B	30	32.45	0.71
TP53	39	90.81	0.83	PPARG	30	30.67	0.69
ESR1	38	191.43	0.82	MMP2	30	20.98	0.72
TNF	37	60.24	0.80	NOS3	29	33.24	0.70
CASP3	37	53.13	0.80	IL10	29	22.41	0.69
MYC	33	31.33	0.75	CXCL8	29	74.95	0.70
SIRT1	33	40.93	0.74	AR	27	145.64	0.69
CTNNB1	32	35.40	0.74	FOXO1	25	18.75	0.66
PTGS2	31	30.75	0.73	IGF1R	24	22.09	0.66
PTEN	31	34.19	0.73				

### Pathway and Functional Enrichment Analysis

**Figure 3.**
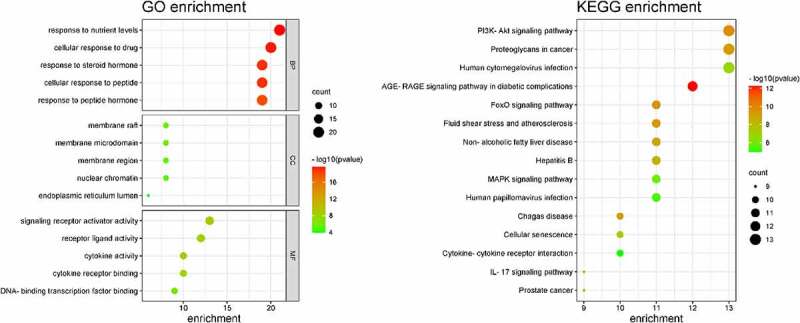
GO enrichment and KEGG enrichment.

**Figure 4.**
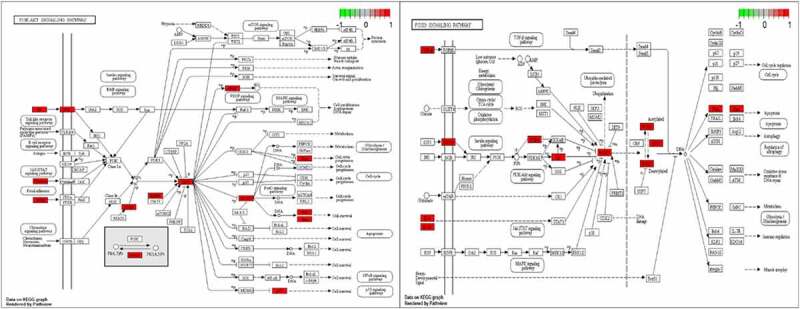
PI3K-AKT pathway diagram and FoxO pathway diagram; The red dots represent the enriched target.

GO enrichment analysis can determine its biological functions related to the fight against POF from the three aspects of biological process (BP), cell component (CC), and molecular function (MF) (see Figure 3). It shows that BBR may interact with the activation of signal receptors, ligands, and cytokines on the cell membrane, so that can participate in regulating the response of cells to drugs, steroid hormones, peptides, and peptide hormones to regulate the level of various hormones and exert its role in improving POF and protecting the ovaries. KEGG enrichment analysis can explore the potential pathways of BBR in the improvement of POF. It shows that the PI3K-AKT signaling pathway has the highest degree of enrichment and the FoxO and AGE-RAGE signaling pathways (see Figure 3). These pathways regulate biological processes such as inflammation, cell circulation, apoptosis, autoimmune regulation, and so on (see Figure 4).

### Drug-target-pathway network

**Figure 5.**
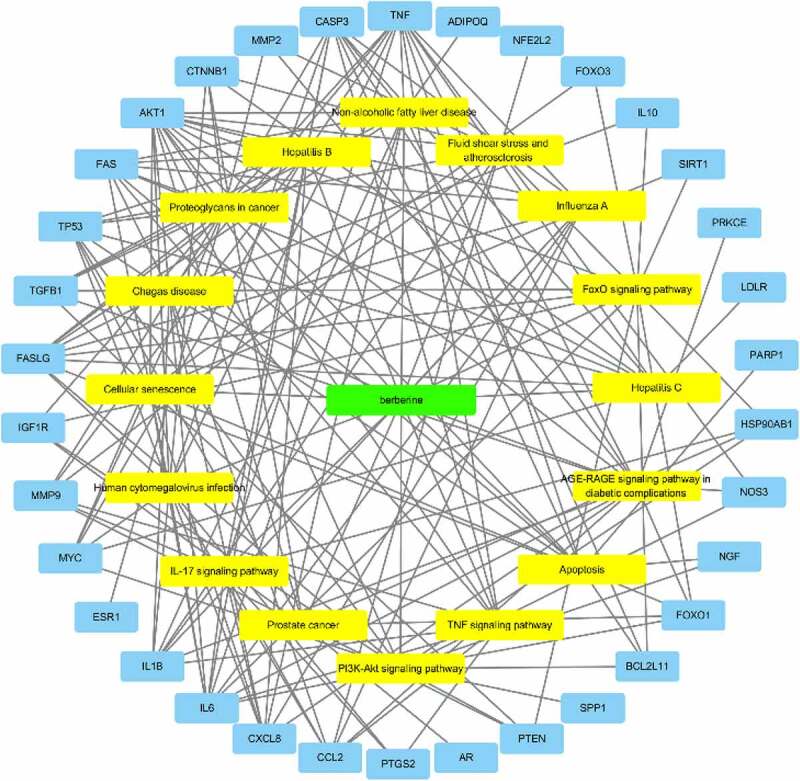
Drug-target-pathway network; The green node represents the drug; the yellow nodes represent the pathways; the blue nodes represent the targets.

Since genes cannot exert their biological and pharmacological effects independently, we established a drug-target-pathway network based on the first 20 signal pathways and related targets and compounds (see Figure 5) to further clarify the molecular mechanism of BBR in the treatment of POF. After integrating drug target prediction, pathway and function enrichment, and network analysis, the PTEN, AKT1, FoxO1, FasL and Bim targets were highly enriched in both PI3K/AKT signaling pathway and FoxO signaling pathway (see Figure 4). They are also considered to be critical targets for the effect of BBR in the improvement of POF. Therefore, we speculate that the development of BBR in improving POF may be involved in regulating cell apoptosis and autoimmunity by regulating the PTEN/AKT1/FoxO1 pathway and Bim and Fas/FasL targets.

## Experimental validation

### Crystal violet staining of vaginal smear

The estrus cycle of rats is generally divided into four periods: proestrus, estrus, postestrus, and anestrus. The proestrus is mainly round or oval nucleated cells, and a small amount of anucleated keratinized epithelial cells can also be seen (see Figure S2A); Irregular polygonal anucleated keratinocytes with an entire field of view can be seen during estrus (see Figure S2B); In the postestrus period, there are mainly a large number of white blood cells and a small number of anucleated keratinocytes (see Figure S2C); Leukocytes, nucleated and non-nucleated cells co-exist during anestrus (see Figure S2D).

### BBR can increase the content of E_2_ and AMH hormones in the serum of rats and reduce the content of FSH and LH hormones

**Figure 6.**
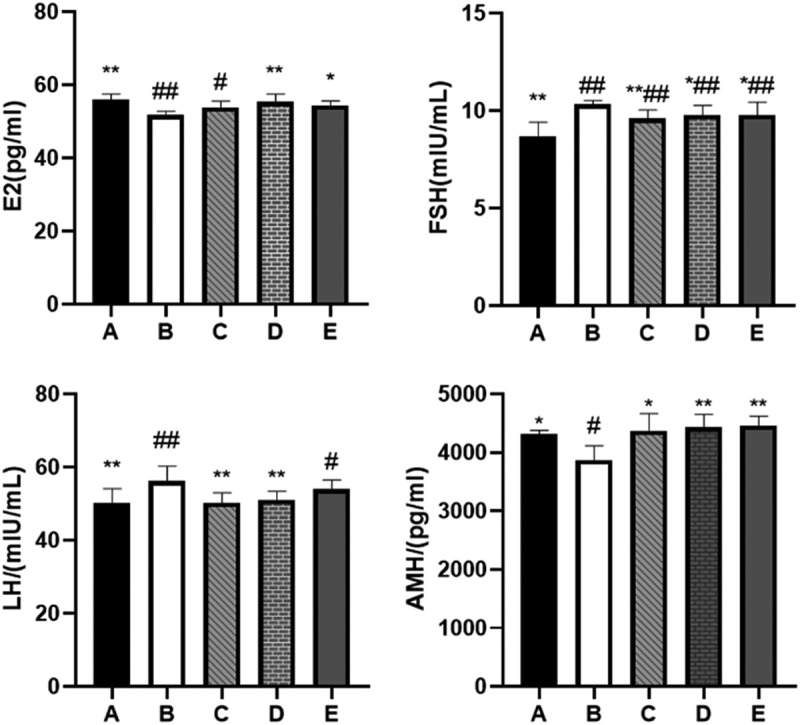
Comparison of serum E2, FSH, LH, AMH content; A: Blank control group, B: Model group, C: Low-dose group, D: Medium-dose group, E: High-dose group; Compared with the model group, *P˂0.05, **P˂0.01; compared with the blank control group, ^#^*P*˂0.05, ^##^*P*˂0.01.

POF can cause abnormal secretion of some hormones in rat serum. We tested 4 hormones in serum that can express ovarian reserve: E_2_, FSH, LH, and AMH. As expected, the serum levels of E_2_ (*P* < 0.01) and AMH (*P* < 0.05) in the POF model rats decreased, and the serum levels of FSH and LH increased (*P* < 0.01) (see Table 3). After 28 days of BBR treatment, the levels of E_2_ and AMH increased to varying degrees. The increase of E_2_ and AMH after treatment with the medium and high dose of BBR was more significant. The levels of FSH and LH in the serum have decreased to varying degrees, among which the hormone reduction after low and medium-dose BBR treatment is more significant (see Figure 6).

**Table 3.** Serum E_2_, FSH, LH, AMH content of rats ($\bar{X}\pm S$)

Group	E_2_/(pg/ml)	FSH/(mIU/mL)	LH/(mIU/mL)	AMH/(pg/ml)
Group A	56.08 ± 1.39**	8.70 ± 0.72**	50.22 ± 3.87**	4309.63 ± 72.74*
Group B	51.77 ± 1.00^##^	10.33 ± 0.18^##^	56.13 ± 4.16^##^	3868.56 ± 246.98^#^
Group C	53.79 ± 1.76^#^	9.59 ± 0.44^**##^	50.19 ± 2.79**	4377.19 ± 285.67*
Group D	55.39 ± 2.11**	9.77 ± 0.49^*##^	51.02 ± 2.32**	4436.38 ± 216.71**
Group E	54.39 ± 1.27*	9.78 ± 0.66^*##^	53.98 ± 2.54^#^	4464.50 ± 160.15**

Note: Group A: Blank control group, Group B: Model group, Group C: Low-dose group, Group D Medium-dose group, Group E: High-dose group; Compared with the model group, *P˂0.05, **P˂0.01; Compared with the blank control group, ^#^*P*˂0.05, ^##^*P*˂0.01.

### Low and medium doses of BBR can increase the ovarian index of rats

**Figure 7.**
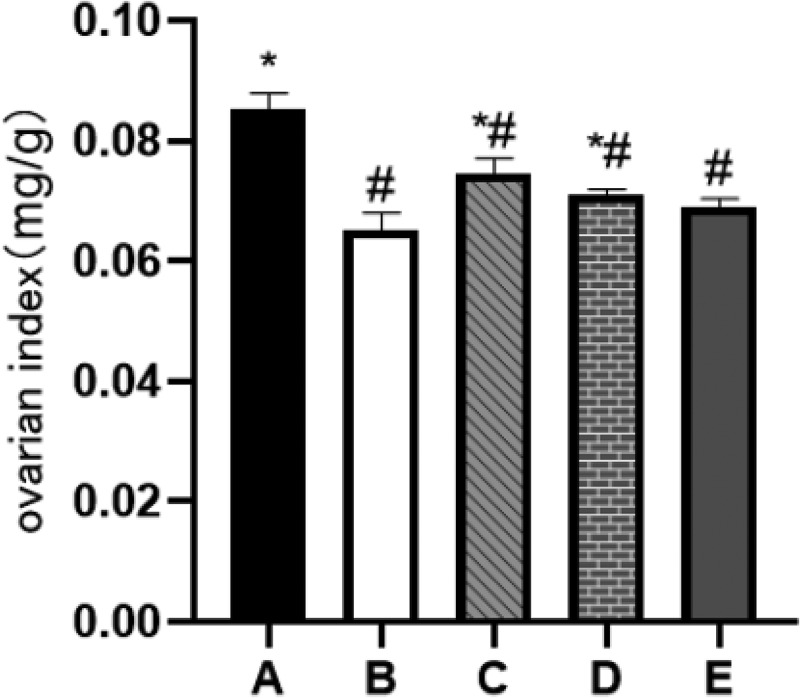
Ovarian index; A: Blank control group, B: Model group, C: Low-dose group, D: Medium-dose group, E: High-dose group; Compared with the model group, *P˂0.05; Compared with the blank control group, ^#^*P*˂0.05.

Ovarian index refers to the ratio of the wet weight of the rat’s bilateral ovaries to the rat’s body weight, which can indicate the health of the ovaries. After measurement, the ovarian index of rats in the POF model group was significantly reduced (*P*˂0.05). After 28 days of continuous intragastric administration of BBR, the ovarian index of rats in the low and medium dose groups increased significantly (*P*˂0.05) (see Figure 7).

### BBR can inhibit the PTEN/AKT1/FoxO1 signaling pathway

**Figure 8.**
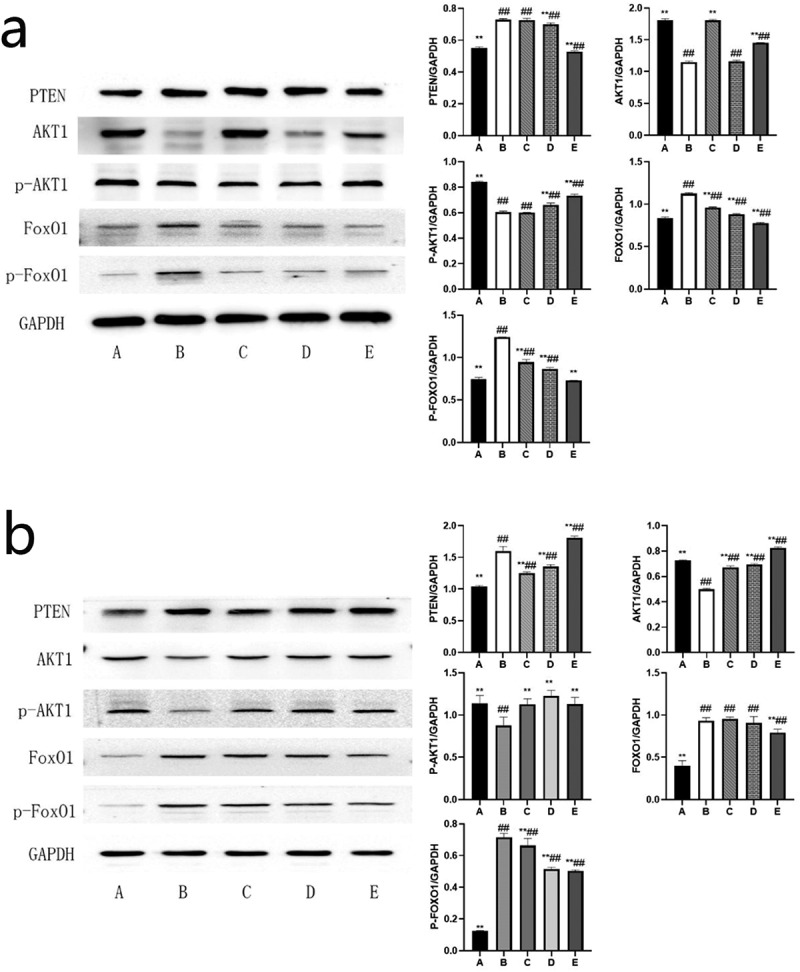
Western blot band and the ratio of the target protein to internal control (normalized processing); (a) The expression level of each target protein in rat ovary; (b) The expression level of each target protein in ovarian granulosa cells; A: Blank control group, B: Model group, C: Low-dose group, D: Medium-dose group, E: High-dose group; Compared with the model group, *P˂0.05, **P˂0.01; Compared with the blank control group, ^#^*P*˂0.05, ^##^*P*˂0.01.

Network pharmacology analysis predicts that the molecular mechanism of BBR’s anti-POF may be highly related to the PTEN/AKT1/FoxO1signaling pathways. Experiments further validated these potential targets and signaling pathways (see Figure 4). The PTEN/AKT1/FoxO1 signaling pathway was activated in the ovaries of rats and GCs in the POF model group. Compared with the blank control group, the PTEN protein content was significantly increased (*P* < 0.01); AKT1 protein content and phosphorylation level were significantly reduced (*P* < 0.01); FoxO1 protein content and its phosphorylation level increased significantly (*P* < 0.01). After the treatment of BBR, the PTEN/AKT1/FoxO1 signaling pathway in the ovaries of rats and GCs in the low, medium, and high dose groups were all inhibited to varying degrees (see Figure 8).

### BBR can inhibit the expression of Fas/FasL and Bim mRNA in the ovaries of rats and GCs

**Figure 9.**
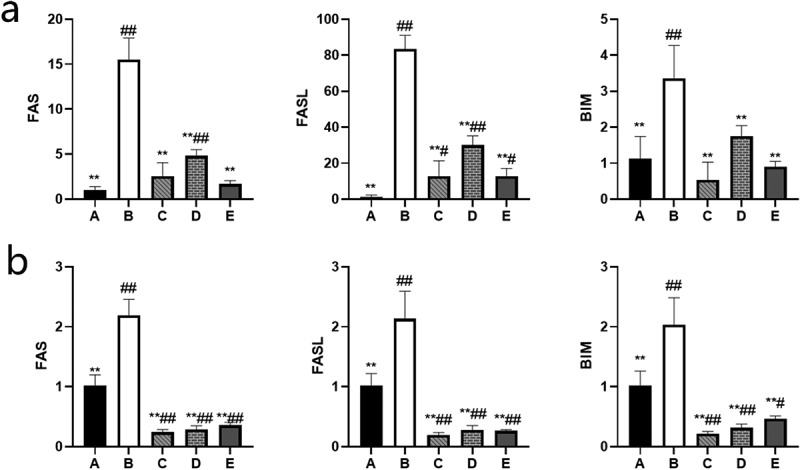
Relative expression levels of target genes in each group; (a) The relative expression of each target gene in rat ovary; (b) The relative expression of each target gene in ovarian granulosa cells; A: Blank control group, B: Model group, C: Low-dose group, D: Medium-dose group, E: High-dose group; Compared with the model group, *P˂0.05, **P˂0.01; Compared with the blank control group, ^#^*P*˂0.05, ^##^*P*˂0.01.

After measurement, Fas/FasL and Bim mRNA were overexpressed in the ovaries of rats and GCs in the POF model group (*P*˂0.01). After the treatment of BBR, the expression of Fas/FasL and Bim mRNA in the ovaries of rats and GCs in the low, medium, and high dose BBR groups were inhibited to varying degrees(*P*˂0.01) (see Figure 9).

### BBR can improve the viability of POF-GCs

**Figure 10.**
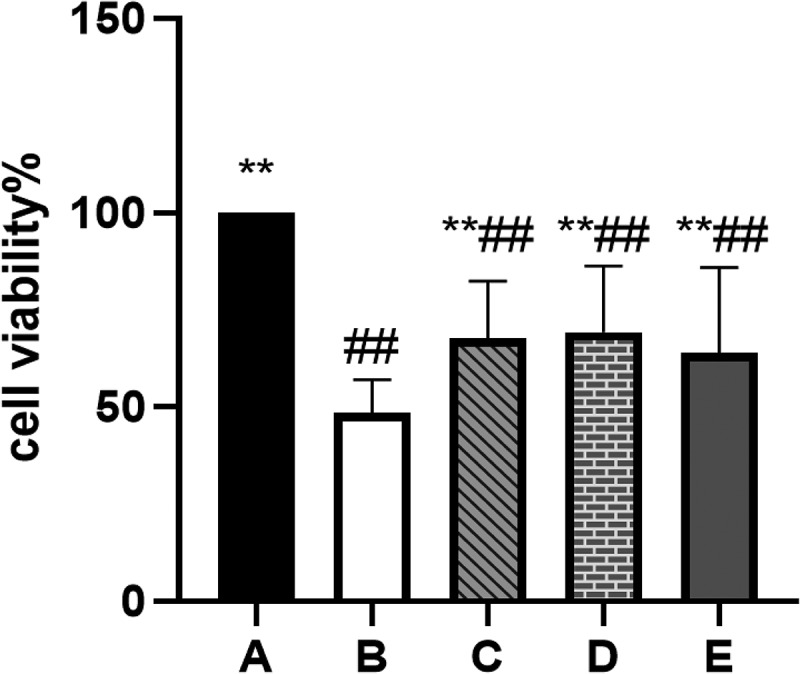
Cell viability determined by CCK8; A: Blank control group, B: Model group, C: Low-dose group, D: Medium-dose group, E: High-dose group; Compared with the model group, *P˂0.05, **P˂0.01; Compared with the blank control group, ^#^*P*˂0.05, ^##^*P*˂0.01.

The CCK-8 shows that the viability of GCs in the POF model group was significantly reduced (*P*˂0.01). The viability of GCs protected by BBR was increased to varying degrees compared with that of the model group (*P*˂0.01) (see Figure 10).

## Discussion

POF is a complex disease that can lead to estrogen deficiency, ovarian failure, and infertility. It has a variety of pathological mechanisms. BBR is an alkaloid extracted from natural medicines. Its effect is widely used in many clinical fields, but its mechanism of action is complex, involving multiple targets and pathways. The unclear mechanism of BBR is still an obstacle to its widespread use. The emergence of network pharmacology provides new methods for investigating the mechanism of action of drugs and diseases. In this study, network pharmacology analysis and in vivo experimental verification were used to explore the potential targets and pathways of BBR in improving POF.

Combining network topology analysis and PPI network analysis, it is found that the drug acts on multiple targets and signal pathways. We have determined the core targets and pathways of the drug’s action: PTEN /AKT, FoxO, Fas/FasL, Bim. These pathways and targets are related to the proliferation of cells, the processes of apoptosis [24], cell cycle, cell death, metabolism, and oxidative stress [25,26]. And these processes play an essential role in the occurrence and development of POF [27–29]. Therefore, we speculate that BBR may improve POF through the combined action of PTEN/AKT1/FoxO1 signaling pathway and Fas/FasL, Bim targets. To verify this hypothesis, we explored the improvement and protection of BBR on POF rats and its molecular mechanism from both in vivo and in vitro experiments.

Estradiol (E_2_) is a sex steroid hormone that can stimulate the proliferation and differentiation of GCs in the growth phase. Follicle-stimulating hormone (FSH) is secreted by the anterior pituitary gland, stimulating follicles to produce estrogen [30]. When the ovarian reserve is reduced, the secretion of inhibin is diminished. It is not enough to make enough inhibin to inhibit the secretion of FSH, which will lead to the phenomenon of high serum FSH and low E_2_. BBR can reduce the level of FSH in the serum and increase the level of E_2_ to improve this phenomenon. The basic FSH level can indirectly reflect the size of the follicular pool. FSH has a high specificity for judging the reduction of ovarian reserve, but the sensitivity is not high [30]. Luteinizing hormone (LH) is a glycoprotein secreted by the anterior pituitary gland, which plays a decisive role in the formation of ovulation [31]. Antral follicles rapidly synthesize E_2_ under the influence of LH, which is essential for oocyte maturation and preparation of the endometrium for implantation. It also needs to activate the hypothalamic-pituitary-ovarian axis, which is used to induce the LH peak required for ovulation [31]; the lack of E_2_ in POF can increase serum LH. BBR can increase E_2_ levels and return LH to normal levels. AMH is a member of the transforming growth factor-β (TGF-β) family. Studies have shown that serum AMH levels are more predictive of POF than FSH or E_2_ levels [32]. And serum AMH is the preferred marker of ovarian reserve capacity [33]. People with insufficient ovarian reserve have significantly lower AMH levels than normal. In vivo experiment shows that different doses of BBR can regulate the levels of serum hormones to varying degrees; Among them, the medium-dose BBR can significantly regulate the levels of the four hormones in the serum, the high-dose BBR has more significant effects on regulating E_2_ and AMH levels. In contrast, the low-dose BBR has more significant effects on regulating FSH and LH levels.

The PI3K/PTEN/AKT signaling pathway regulates the inhibition and activation of primordial follicles, and the balance in this pathway is regulated by PTEN, which is essential for maintaining the dormant state of primordial follicle reserves [34]. Studies have confirmed that PTEN can negatively regulate the PI3K/AKT pathway to affect cell proliferation and apoptosis [24] and induce cell cycle arrest in the G1 phase [35]. Mammalian oocytes are the programming headquarters for follicular activation. Oocytes can control the initiation of oocyte growth through the PTEN/PI3K pathway to control the activation of follicles [36,37]. Prematurely activated follicles are atresia in early adulthood (about 3 months old), leading to POF. Studies have shown that curcumin can maintain the stability of ovarian reserve by regulating the PTEN/AKT pathway [38]. Overexpression of PTEN can reverse the activation of primordial follicles, leading to atresia [39]. GCs apoptosis is the primary mechanism of follicular atresia at different developmental stages. GCs provide necessary growth signals and nutrients for oocyte maturation. The state of GCs is critical to follicular development [7]. The PTEN gene in GCs may affect FSH’s follicular and follicular development regulation by regulating the phosphorylation of AKT and FoxO1 [40]. PTEN can also activate the protein kinase B or AKT (PKB/AKT) pathway, increase the growth and activation of primordial follicles [41], lead to the growth of antral follicles [42] and induce cell growth and proliferation, and inhibit cell apoptosis [24]. Cytotoxic drugs can significantly increase PTEN to reduce AKT1, cause GCs apoptosis, and increase POF risk. Since the AKT1 and Hippo signaling pathways can act synergistically to regulate the growth of follicles, the induced mutation of the PTEN/AKT1 gene can accelerate the rate of ovarian reserve failure and lead to POF [43]. FoxO protein is an important downstream target of the PI3K/AKT signal axis and a regulator of the cell cycle, death, metabolism, and oxidative stress [25]. Studies have shown that the activation of phosphorylation of AKT in vivo and the inhibition of phosphorylation of FoxO1 may be related to the survival and proliferation of GCs [44] and the increase in the number of follicles in the pre-ovulatory stage [40]. It can be seen that PTEN/AKT1/FoxO1 is very important for the treatment of POF. Our research shows that the PTEN /AKT1/FoxO1 pathway in the ovaries of rats with POF is overactivated. Different doses of BBR treatment have different levels of inhibition of this pathway, which proves that BBR may inhibit the PTEN /AKT1/FoxO1 pathway to treat POF. The homeostasis regulated by Bim is necessary for normal cell death and survival. The precise regulation of Bim expression has been proved to be an essential condition for the normal development of cells [45]. Bim has pro-apoptotic activity and can be regulated at multiple levels. (FoxO) family proteins can up-regulate Bim transcription [46]. Studies have shown that Bim is an essential mediator of apoptosis in response to FoxO3 activation. The FoxO3a transcription factor induces Bim. AKT directly phosphorylates FoxO3a to prevent it from activating Bim transcription [47,48]. Therefore, BBR may regulate AKT/FoxO to inhibit the expression of Bim. The most common proteins that cause GCs apoptosis in the death receptor pathway include FasL and Fas. Fas/FasL can also induce apoptosis in vitro [26]. Studies have also shown that Fas/FasL can significantly inhibit the proliferation of bovine follicular GCs [28]. It can be seen that Bim and Fas/FasL are both apoptosis-related factors. Our research shows that Bim and Fas/FasL mRNA are overexpressed in the ovaries of rats with POF, and after BBR treatment, the expression of Bim and Fas/FasL mRNA is inhibited varying degrees. We can speculate that BBR can improve POF by inhibiting the expression of Bim and Fas/FasL mRNA.

In this study, ovarian index and serum endohormone level were used as indicators to evaluate ovarian function, but both ovarian index and hormone level were indirect evidence to prove the improvement of ovarian function. Histological analysis of the ovaries comparing the size of the ovaries and the number of follicles provides direct evidence of improved ovarian function. However, due to some objective factors, histological study cannot be carried out, so this study can only indirectly prove that ovarian function has improved, which is the limitation of this experiment.

## Conclusion

This study uses a combination of network pharmacological analysis and experimental verification to explore the effect of BBR on improving POF and its molecular mechanism. Through network pharmacology data mining and in vivo experimental validation, we proved that BBR could inhibit ovarian GCs apoptosis and follicular atresia and regulate E_2_, FSH, LH, AMH hormone secretion by inhibiting the PTEN/AKT1/FoxO1 signaling pathway and reducing the expression of Bim and Fas/FasL mRNA so that can increase ovarian reserve capacity, exert its anti-POF effect. The combination of network pharmacology prediction and in vivo experimental verification can effectively clarify the multi-target and multi-path pharmacological action mechanism of naturally extracted drugs.

## Supplementary Material

Supplemental Material

## Data Availability

The data that support this study are available from the corresponding author upon reasonable request.

## References

[1] Goswami D, Conway GS. Premature ovarian failure. Hum Reprod Update. 2005;11(4):391–410. 15919682 10.1093/humupd/dmi012

[2] LJTNEjom N. Clinical practice. Primary ovarian insufficiency. 2009;360(6):606–614. 10.1056/NEJMcp0808697 PMC2762081 19196677

[3] Trabert B, Wentzensen N, Yang HP, et al. Ovarian cancer and menopausal hormone therapy in the NIH-AARP diet and health study. Br J Cancer. 2012;107(7):1181–1187. 22929888 10.1038/bjc.2012.397 PMC3461172

[4] Trabert B, Wentzensen N, Yang HP, et al. Is estrogen plus progestin menopausal hormone therapy safe with respect to endometrial cancer risk? Int J Cancer. 2013;132(2):417–426. 22553145 10.1002/ijc.27623 PMC3427719

[5] Kuang H, Duan Y, Li D, *et al*. The role of serum inflammatory cytokines and berberine in the insulin signaling pathway among women with polycystic ovary syndrome. PLoS One. 2020;15(8):e0235404. 32785222 10.1371/journal.pone.0235404 PMC7423132

[6] Zhang N, Liu X, Zhuang L, et al. Berberine decreases insulin resistance in a PCOS rats by improving GLUT4: dual regulation of the PI3K/AKT and MAPK pathways. Regul Toxicol Pharmacol. 2020;110:104544. 31778716 10.1016/j.yrtph.2019.104544

[7] Liu M, Qiu Y, Xue Z, et al. Small extracellular vesicles derived from embryonic stem cells restore ovarian function of premature ovarian failure through PI3K/AKT signaling pathway. Stem Cell Res Ther. 2020;11(1):3. 31900201 10.1186/s13287-019-1508-2 PMC6942273

[8] Luo TT, Lu Y, Yan SK, et al. Network Pharmacology in Research of Chinese Medicine Formula: methodology, Application and Prospective. Chin J Integr Med. 2020;26(1):72–80. 30941682 10.1007/s11655-019-3064-0

[9] Ru J, Li P, Wang J, *et al*. TCMSP: a database of systems pharmacology for drug discovery from herbal medicines. J Cheminform. 2014;6(1):13. 24735618 10.1186/1758-2946-6-13 PMC4001360

[10] Szklarczyk D, Santos A, von Mering C, et al. STITCH 5: augmenting protein-chemical interaction networks with tissue and affinity data. Nucleic Acids Res. 2016;44(D1):D380–384. 26590256 10.1093/nar/gkv1277 PMC4702904

[11] Stelzer G, Rosen N, Plaschkes I, *et al*. The GeneCards Suite: from Gene Data Mining to Disease Genome Sequence Analyses. Curr Protoc Bioinformatics. 2016;54(1):1 30 31–31 30 33. 27322403 10.1002/cpbi.5

[12] Szklarczyk D, Gable AL, Lyon D, *et al*. STRING v11: protein-protein association networks with increased coverage, supporting functional discovery in genome-wide experimental datasets. Nucleic Acids Res. 2019;47(D1):D607–D613. 30476243 10.1093/nar/gky1131 PMC6323986

[13] Shannon P, Markiel A, Ozier O, et al. Cytoscape: a software environment for integrated models of biomolecular interaction networks. Genome Res. 2003;13(11):2498–2504. 14597658 10.1101/gr.1239303 PMC403769

[14] The Gene Ontology C. The Gene Ontology Resource: 20 years and still GOing strong. Nucleic Acids Res. 2019;47(D1):D330–D338. 30395331 10.1093/nar/gky1055 PMC6323945

[15] Kanehisa M, Furumichi M, Sato Y, et al. KEGG: integrating viruses and cellular organisms. Nucleic Acids Res. 2021;49(D1):D545–D551. 33125081 10.1093/nar/gkaa970 PMC7779016

[16] Meirow D, Lewis H, Nugent D, et al. Subclinical depletion of primordial follicular reserve in mice treated with cyclophosphamide: clinical importance and proposed accurate investigative tool. Hum Reprod. 1999;14(7):1903–1907. 10402415 10.1093/humrep/14.7.1903

[17] Marcondes FK, Bianchi FJ, Tanno AP. Determination of the estrous cycle phases of rats: some helpful considerations. Braz J Biol. 2002;62(4A):609–614. 12659010 10.1590/s1519-69842002000400008

[18] Hornbeck PV. Enzyme-Linked Immunosorbent Assays. Curr Protoc Immunol. 2015;110(1):2 1 1–2 1 23. 26237010 10.1002/0471142735.im0201s110

[19] Hirano S. Western blot analysis. Methods Mol Biol. 2012;926:87–97. 22975958 10.1007/978-1-62703-002-1_6

[20] Jozefczuk J, Adjaye J. Quantitative real-time PCR-based analysis of gene expression. Methods Enzymol. 2011;500:99–109. 21943894 10.1016/B978-0-12-385118-5.00006-2

[21] Zachow R, Uzumcu M. The methoxychlor metabolite, 2,2-bis-(p-hydroxyphenyl)-1,1,1-trichloroethane, inhibits steroidogenesis in rat ovarian granulosa cells in vitro. Reprod Toxicol. 2006;22(4):659–665. 16737795 10.1016/j.reprotox.2006.04.018

[22] Odell ID, Cook D. Immunofluorescence techniques. J Invest Dermatol. 2013;133(1):e4. 10.1038/jid.2012.455 23299451

[23] Qin C, Zhang Q, Wu G. RANBP9 suppresses tumor proliferation in colorectal cancer. Oncol Lett. 2019;17(5):4409–4416. 30988811 10.3892/ol.2019.10134 PMC6447939

[24] Fu X, He Y, Wang X, et al. Overexpression of miR-21 in stem cells improves ovarian structure and function in rats with chemotherapy-induced ovarian damage by targeting PDCD4 and PTEN to inhibit granulosa cell apoptosis. Stem Cell Res Ther. 2017;8(1):187. 28807003 10.1186/s13287-017-0641-z PMC5556338

[25] Burgering BM, Kops GJ. Cell cycle and death control: long live Forkheads. Trends Biochem Sci. 2002;27(7):352–360. 12114024 10.1016/s0968-0004(02)02113-8

[26] Cai L, Zong DK, Tong GQ, et al. Apoptotic mechanism of premature ovarian failure and rescue effect of Traditional Chinese Medicine: a review. J Tradit Chin Med. 2021;41(3):492–498. 34114409 10.19852/j.cnki.jtcm.2021.03.017

[27] Ishise S, Okuda K, Kumazawa T, et al. Proceedings: rupture of Valsalva’s sinus –a case study. Jpn Circ J. 1975;39(6):739. 1152257

[28] Yang R, Xu S, Zhao Z, et al. Fas ligand expression and mediated activation of an apoptosis program in bovine follicular granulosa cells. Gene. 2012;493(1):148–154. 22155318 10.1016/j.gene.2011.11.032

[29] Jang H, Lee OH, Lee Y, *et al*. Melatonin prevents cisplatin-induced primordial follicle loss via suppression of PTEN/AKT/FOXO3a pathway activation in the mouse ovary. J Pineal Res. 2016;60(3):336–347. 26882203 10.1111/jpi.12316

[30] Jamil Z, Fatima SS, Ahmed K, et al. Anti-Mullerian Hormone: above and Beyond Conventional Ovarian Reserve Markers. Dis Markers. 2016;2016:5246217. 26977116 10.1155/2016/5246217 PMC4764725

[31] Weghofer A, Schnepf S, Barad D, et al. The impact of luteinizing hormone in assisted reproduction: a review. Curr Opin Obstet Gynecol. 2007;19(3):253–257. 17495642 10.1097/GCO.0b013e3280bad843

[32] Visser JA, Schipper I, Laven JS, et al. Anti-Mullerian hormone: an ovarian reserve marker in primary ovarian insufficiency. Nat Rev Endocrinol. 2012;8(6):331–341. 22231848 10.1038/nrendo.2011.224

[33] Moolhuijsen LME, Visser JA. Anti-Mullerian Hormone and Ovarian Reserve: update on Assessing Ovarian Function. J Clin Endocrinol Metab. 2020;105(11):3361–3373. 10.1210/clinem/dgaa513 PMC7486884 32770239

[34] Kalich-Philosoph L, Roness H, Carmely A, et al. Cyclophosphamide triggers follicle activation and “burnout”; AS101 prevents follicle loss and preserves fertility. Sci Transl Med. 2013;5(185):185ra162. 10.1126/scitranslmed.3005402 23677591

[35] Yang M, Lin L, Sha C, *et al*. Bone marrow mesenchymal stem cell-derived exosomal miR-144-5p improves rat ovarian function after chemotherapy-induced ovarian failure by targeting PTEN. Lab Invest. 2020;100(3):342–352. 31537899 10.1038/s41374-019-0321-y

[36] Reddy P, Liu L, Adhikari D, *et al*. Oocyte-specific deletion of Pten causes premature activation of the primordial follicle pool. Science. 2008;319(5863):611–613. 18239123 10.1126/science.1152257

[37] Adhikari D, Liu K. Molecular mechanisms underlying the activation of mammalian primordial follicles. Endocr Rev. 2009;30(5):438–464. 19589950 10.1210/er.2008-0048

[38] Lv Y, Cao RC, Liu HB, et al. Single-Oocyte Gene Expression Suggests That Curcumin Can Protect the Ovarian Reserve by Regulating the PTEN-AKT-FOXO3a Pathway. Int J Mol Sci. 2021;22(12):6570. 34207376 10.3390/ijms22126570 PMC8235657

[39] Hu Y, Yuan DZ, Wu Y, *et al*. Bisphenol A Initiates Excessive Premature Activation of Primordial Follicles in Mouse Ovaries via the PTEN Signaling Pathway. Reprod Sci. 2018;25(4):609–620. 28982275 10.1177/1933719117734700

[40] Fan HY, Liu Z, Cahill N, et al. Targeted disruption of Pten in ovarian granulosa cells enhances ovulation and extends the life span of luteal cells. Mol Endocrinol. 2008;22(9):2128–2140. 18606860 10.1210/me.2008-0095 PMC2631369

[41] Kallen A, Polotsky AJ, Johnson J. Untapped Reserves: controlling Primordial Follicle Growth Activation. Trends Mol Med. 2018;24(3):319–331. 29452791 10.1016/j.molmed.2018.01.008 PMC6482944

[42] Wesevich V, Kellen AN, Pal L. Recent advances in understanding primary ovarian insufficiency. F1000Res. 2020;9:1101. 10.12688/f1000research.26423.1 PMC7477642 32934798

[43] Monniaux D, Clement F, Dalbies-Tran R, et al. The ovarian reserve of primordial follicles and the dynamic reserve of antral growing follicles: what is the link? Biol Reprod. 2014;90(4):85. 24599291 10.1095/biolreprod.113.117077

[44] Park Y, Maizels ET, Feiger ZJ, et al. Induction of cyclin D2 in rat granulosa cells requires FSH-dependent relief from FOXO1 repression coupled with positive signals from Smad. J Biol Chem. 2005;280(10):9135–9148. 15613482 10.1074/jbc.M409486200 PMC1564190

[45] Yue D, Sun X. Idelalisib promotes Bim-dependent apoptosis through AKT/FoxO3a in hepatocellular carcinoma. Cell Death Dis. 2018;9(10):935. 30224718 10.1038/s41419-018-0960-8 PMC6141589

[46] Shukla S, Saxena S, Singh BK, et al. BH3-only protein BIM: an emerging target in chemotherapy. Eur J Cell Biol. 2017;96(8):728–738. 29100606 10.1016/j.ejcb.2017.09.002

[47] Stahl M, Dijkers PF, Kops GJ, et al. The forkhead transcription factor FoxO regulates transcription of p27Kip1 and Bim in response to IL-2. J Immunol. 2002;168(10):5024–5031. 11994454 10.4049/jimmunol.168.10.5024

[48] Harada H, Grant S. Targeting the regulatory machinery of BIM for cancer therapy. Crit Rev Eukaryot Gene Expr. 2012;22(2):117–129. 22856430 10.1615/critreveukargeneexpr.v22.i2.40 PMC3834587

