# Supplementary material for: Research and experimental verification of the molecular mechanism of berberine in improving premature ovarian failure based on network pharmacology
Source: Bioengineered. 2022 Apr 14;13(4):9885–900. doi: 10.1080/21655979.2022.2062104 (PMC9161839; doi:10.1080/21655979.2022.2062104)
Supplement: Supplemental Material [file KBIE_A_2062104_SM4760.zip › Supplemental material/Figure S1.pdf]

锦州医科大学实验动物福利与伦理审查表

Jinzhou Medical University Application for Laboratory Animal Welfare and Ethical Review

申请日期：2021.9.6　　批准日期：2021.9.8　　批准文号：2021016

Appl. Date：2021.9.6　　Appr. Date：　　Issue No.

一、项目与人员信息					
课题名称及编号 Program and No	中文 基于网络药理学和体内实验探讨黄连素治疗卵巢早衰的分子机制<br>英文（下同）Explore the molecular mechanism of BBR in the treatment of POF based on network pharmacology and in vivo experiments				
课题负责人 Name of rincipal Investigator	艾浩 Ai Hao	课题来源 Sponsor	卵泡生殖发育重点实验室 Laboratory of Follicle Reproductive Development		
单位/科室 Department	锦州医科大学 Jinzhou Medical University	电 话 Tel. No	~~[illegible]~~ 18842674994	信箱 E-mail	wuxueman@foxmail.com
动物实验负责人 Contact Person	吴雪 Wu Xue	电 话 Tel. No	18853149994	信箱 E-mail	1271070065@qq.com

参与动物实验操作人员姓名、培训编号　Name and certificate number

姓 名 Name	单位/科室 Department	是否经过培训 License Yes or No	电话 Tel. No	信箱 E-mail
吴雪 Wu Xue	锦州医科大学 Jinzhou Medical University	是	18853149994	1271070065@qq.com.

二、实验动物信息			
动物来源 Animal origin	☑实验动物中心 Department of Laboratory Animals<br>□采购/赠予(Procurement / Gift)<br>（具体单位名称：__________）<br>□ 国外引进(International purchase)	质量合格证 Certification of fitness	☑有 Yes<br>□ 无 No
品种/品系 breed/strain	☑大鼠(Rat) SD<br>□小鼠(Mouse)______<br>□ 豚鼠(Guinea pig)____ □ 兔(rabbit) ____<br>□ 犬 (Dog)______ □ 猪(Pig) ______<br>□ 猴（Monkey）____ □其它（Other） ____	动物级别 Grade	□普通(CV)<br>□清洁(CL)<br>☑SPF<br>□无菌动物（GF）

数量（只） Number (♀; ♂)	雌（♀） 30 ;<br>雄（♂） ____ ;	周/月龄 W/M Age	5周 5w	体重（g） Weight	130g~150g

拟实验时间：
Experimental period：2021.9 ~ 2022.4

三、研究项目信息

1. 动物实验项目的目的、必要性、意义和如何设计以达成研究目标
   Experimental objective, necessity and significance and how the program has been designed to achieve the objectives of the research

   探究治疗卵巢早衰的新疗法及其机制，为卵巢早衰的治疗提供新支持。
   使用网络药理学预测与体内实验相结合的方法。
   Explore new therapies and mechanisms for POF. Provide new support for the treatment of POF. Using a combination of network pharmacological prediction and in vivo experimental verification.

2. 说明实验对动物可能造成的所有可预期的伤害，包括每个实验方案中可能产生副作用的细节以及采取的防控措施
   Description of the overall harms expected to be experienced by the animals – including details of the likely adverse effects of each protocol and the steps which will be taken to control these adverse effects

   ①长期注射药物可能导致动物皮肤的损害：将腹部分区，在不同分区注射
   ②灌胃造成损害：提升实验人员操作水平
   ① Long-term injection of drugs may cause damage to the skin of animals: divide the abdomen into different zones for injection ②Damage caused by gastric gavage: improve the experimenter's operation level.

3. 主要观察指标
   Main observation target

   ① 阴道涂片 ② 血清激素水平
   ③ 蛋白表达量 ④ 目的DNA含量
   ① Vaginal smear. ② Serum hormone levels ③ Protein content
   ④ DNA content

4. 仁慈终点或实验终结的指标
   Humane endpoint or experimental terminative indicator

   黄连素连续治疗28天后。
   After 28 days of BBR treatment

5. 动物死亡处理
   Death conduct

   - ☐ CO_2窒息 CO_2 suffocated
   - ☐ 麻醉后放血致死 Exsanguinations with anesthesia
   - ☐ 颈椎脱臼致死 Cervical dislocation
   - ☑ 麻醉过量致死 Anesthesia overdose
   - ☐ 其他 Others, detailed description

   请详细说明如下： 治疗结束后，苯巴比妥过量处死
   After treatment. phenobarbital overdose was executed

6. 非处死动物的处置方式
   Not for the death of the animal disposition

   ☐ 继续使用 ☐ 保存的机构

Continue to use　　　　　　Save in the agency

□ 放生野外　　　　　　　　□ 其他，详细说明

Release to the wild　　　　　Others, detailed description

7. 动物替代、减少动物用量、降低动物痛苦伤害的主要措施

Major measure for 3Rs 动物两个卵巢分别用于WB和PCR. 减少动物的使用. 保障动物的生存质量. 操作轻柔.

The two ovaries of rats are used for WB and PCR respectively. reducing the use of animals. Guarantee the quality of life of animals. the operation is gentle.

8. 是否使用有毒（害）物质 （感染、放射、化学毒、其他）

Poisonous (harmful) material(infection, radiate, chemical poison and other)being used

☑ 是　　　　□否

Yes　　　　no

说明：使用化疗药物环磷酰胺造模

Declare Modeling with the chemotherapy drug cyclophosphamide.

四、 有关福利伦理审查的补充说明(若没有请填写无)

Supplementary instruction for investigate

无. None

五、 信息公开和保密要求：说明哪些信息需要保密，哪些信息可以公开

Declaration for the information disclosure and confidentiality requirements, declaring the information need to be kept secret ,the information can be disclosed

所有信息可公开

All information can be made public.

六、 对伦理审查委员有无回避要求(若没有请填写无)

Claiming jurors for being debarb

无 None

**声明：**<br>1. 我将自觉遵守实验动物福利伦理相关法规和各项规定，同意接受伦理委员会和实验动物室管理者的监督与检查；<br>2. 本人保证本申请表中所填内容真实、详尽和易懂。<br>Declaration：<br>1. I will abide by the law and regulation stipulation, and accept the supervision and inspection by the committee and laboratory animal department.<br>2. The information I have given is accurate, detailed and comprehensive.<br><br>声明人：课题负责人签（章） [illegible]<br>Declarant：Signature (stamp) of PI<br>动物实验负责人签（章） [illegible]<br>Signature (stamp) of Director of animal experiment<br>2021 年 9 月 6 日
申报单位审批意见<br>Approval opinion<br><br>☑ 批准 Approval　　☐ 不批准 Not approve<br><br>指定负责人签（章）：[illegible]<br>Authorized Personnel Signature（Stamp）<br>2021 年 9 月 8 日
实验动物伦理委员会审批意见<br>Approval opinion<br><br>徐州医科大学实验动物伦理委员会<br>主任委员签（章）：<br>年 月 日
