# Supplementary material for: Research and experimental verification of the molecular mechanism of berberine in improving premature ovarian failure based on network pharmacology
Source: Bioengineered. 2022 Apr 14;13(4):9885–900. doi: 10.1080/21655979.2022.2062104 (PMC9161839; doi:10.1080/21655979.2022.2062104)
Supplement: Supplemental Material [file KBIE_A_2062104_SM4760.zip › Supplemental material/Figure S2.pdf]

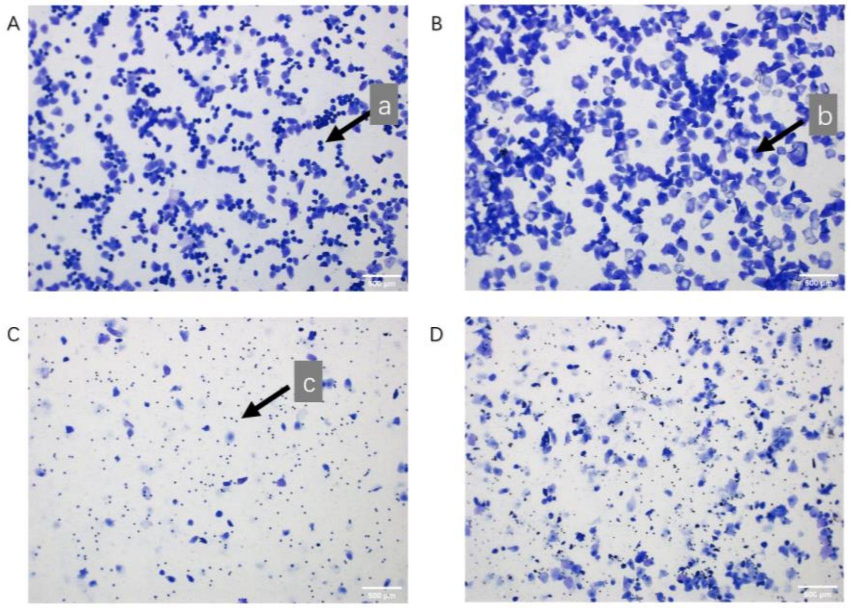

Figure S2. Crystal Violet Staining of Vaginal Smear; A: Proestrus (×100); B: Estrus (×100); C: Postestrus (×100); D: Anestrus (×100); a: Nucleated cells; b: Anucleated keratinized epithelial cells; c: White blood cells.
